# Extremely low daylight sea-crossing flights of a nocturnal migrant

**DOI:** 10.1093/pnasnexus/pgad225

**Published:** 2023-07-08

**Authors:** Gabriel Norevik, Susanne Åkesson, Anders Hedenström

**Affiliations:** Centre for Animal Movement Research, Department of Biology, Lund University, 223 62 Lund, Sweden; Centre for Animal Movement Research, Department of Biology, Lund University, 223 62 Lund, Sweden; Centre for Animal Movement Research, Department of Biology, Lund University, 223 62 Lund, Sweden

**Keywords:** bird migration, ecological barrier, flight cost, flight behavior, altitude

## Abstract

Understanding the trade-off between energy expenditure of carrying large fuel loads and the risk of fuel depletion is imperative to understand the evolution of flight strategies during long-distance animal migration. Global flyways regularly involve sea crossings that may impose flight prolongations on migrating land-birds and thereby reduce their energy reserves and survival prospects. We studied route choice, flight behavior, and fuel store dynamics of nocturnally migrating European nightjars (*Caprimulgus europaeus*) crossing water barriers. We show that barrier size and groundspeed of the birds influence the prospects of extended daylight flights, but also that waters possible to cross within a night regularly result in diurnal flight events. The nightjars systematically responded to daylight flights by descending to about a wingspan's altitude above the sea surface while switching to an energy-efficient flap-glide flight style. By operating within the surface–air boundary layer, the nightjars could fly in ground effect, exploit local updraft and pressure variations, and thereby substantially reduce flight costs as indicated by their increased proportion of cheap glides. We propose that surface-skimming flights, as illustrated in the nightjar, provide an energy-efficient transport mode and that this novel finding asks for a reconsideration of our understanding of flight strategies when land-birds migrate across seas.

**Significance Statement**

Extended waters where safe landing is not possible act as ecological barriers for migrating land-birds worldwide, influencing individual energy budgets and shaping migratory flyways. By combining long-term citizen science and bird ringing data with novel, individual-based microdataloggers, we demonstrate how a nocturnal avian migrant systematically descends at dawn to proceed sea-crossing flights just above the surface. This novel behavioral response to an extended flight over open water likely reduces transport costs considerably and therefore alleviates the negative fitness effects associated with water barriers.

## Introduction

Active flight allows avian migrants to perform continental-wide roundtrip movements within the annual cycle to exploit seasonally shifting resources (1). Powering the flight muscles is, however, demanding and the work rate increases steeply with increased (fuel) loads. Thus, an efficient migrant should avoid excessive energy stores that are costly to carry and take time to replenish (2). Small energy stores on the other hand come with the risk of fuel depletion, which may be fatal if safe landing is not possible. Balancing between these costs is a challenge that billions of migrating land-birds face when crossing large water bodies, such as the Gulf of Mexico and the Mediterranean Sea, as well as the Atlantic, Indian, and Pacific Oceans (3–6). How terrestrial migrants respond when facing overwater flights and what drivers underpin their decisions remain to be understood. Assuming that they minimize energy expenditure during long-haul flights, migrants may perform behavioral adjustments by, for example, altering flight mode due to changes in external factors that influence the flight budget. Such in-flight behavior adjustment has been observed in facultative soaring migrants that shift between active flight and cross-country soaring depending on convective thermal conditions (7, 8). Like facultative soarers over land, migrants that primarily use active flight may change to a more energy-efficient flap-gliding flight style when facing extended flights over open water where landing is not possible (9).

Here, we present data on migratory sea crossings by European nightjars *Caprimulgus europaeus* (henceforth nightjar), an aerial insectivorous bird that migrates annually between its Euro–Asian breeding range and wintering areas in southern Africa (10). Nightjars are crepuscular and nocturnal birds that usually remain inactive during daytime. This circadian rhythm is generally maintained during their seasonal migrations, even when passing ecological barriers such as the Sahara Desert (11). However, diurnal flights occur when these terrestrial birds cross large water bodies where landfall is not possible. Previous studies of satellite-tracked birds of other African–European terrestrial migrants have associated such barrier crossings with elevated mortality risks (12, 13), but to our knowledge, no data on where and when nightjars perish during the annual cycle have been collected systematically. For smaller birds, annual recapture rates of previously trapped and ringed birds are often used as proxy for the fraction of the population that survived the nonbreeding season (including migration). Annual recapture rates of previously trapped and ringed nightjars within our study population are about 25%, which is concordant to reports from African–European long-distance migratory passerines (14–16). Although recapture rates likely underestimate actual survival rate and the fraction of returning (but not trapped) birds, it indicates the risks long-distance birds face outside the breeding season (17).

We integrated citizen science data with data generated by microdataloggers to detail how nightjars traverse large barriers. We compiled diurnal observations of migrating birds from citizen science databases to explore the occurrence and causes of extended sea-crossing flights by nightjars. We then asked to what degree nightjars may use proactive behaviors (such as fueling) and reactive (flight) responses when exposed to the potential risks associated with flight across extensive water bodies. We addressed these questions by tracking the 3D flight paths of nightjars during sea crossings and by examining body mass data of birds intercepted just after completing a flight across a large water body. To characterize flight routes, we recorded 128 sea-crossing flights of 26 individuals using pin-point global positioning system (GPS) tags (PathTrack Ltd, Otley, West Yorkshire, UK). To study activity (wing flapping) and flight behavior (vertical movement), we recorded 85 sea-crossing flights of 18 birds using custom-made multisensor dataloggers (MDL) to record vertical acceleration (flapping or flap-gliding) and ambient pressure (flight altitude).

## Results and discussion

**Fig. 1.**
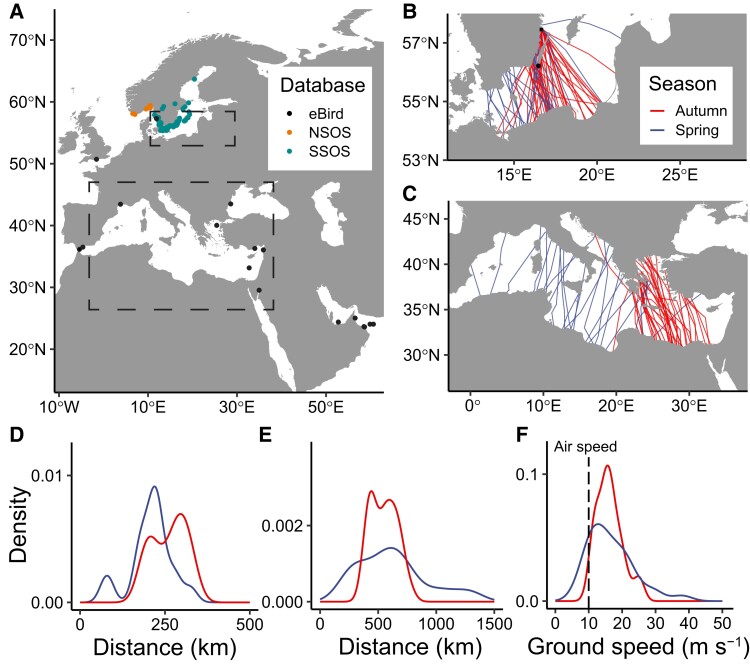
Observations and GPS tracks of migrating nightjars during sea-crossing flights. A) Spatial distribution of 493 observations of nightjars over open water retrieved from three public citizen science databases: eBird, the NSOS, and the SSOS. Dashed rectangles illustrate the location and graphical sections of B) and C). B, C) Overview of 128 GPS tracks across the Baltic and Mediterranean Seas. D, E) Distributions of OWD along GPS tracks over the Baltic D) and Mediterranean E) Seas (note different scales in the *x*-axes). F) Distributions (with GPS tracks over the Baltic and Mediterranean Seas pooled) of ground speed (calculated as the distance between the two GPS locations divided by the 2-h sampling interval) compared with a nightjar's airspeed of 10 m s^−1^ (dashed vertical line). Recorded ground speeds below 10 m s^−1^ indicate head wind, and speeds higher than this indicate tailwind. Colors in B–F) represent autumn (red) and spring (blue).

**Fig. 2.**
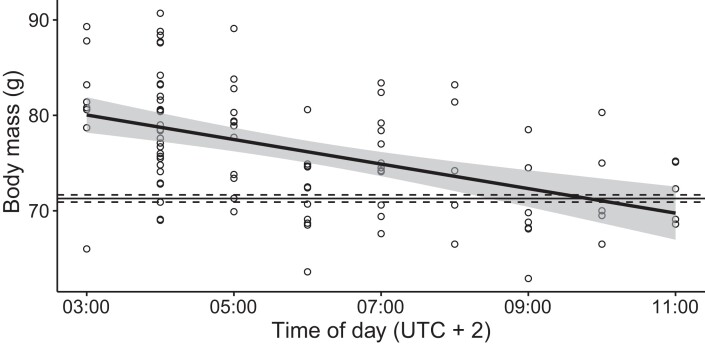
Body mass data of 95 nightjars trapped in SE Sweden after crossing the Baltic Sea in spring relative to time of day. The temporal reduction in body mass is described as a linear regression: Body mass (g) = 83.9 (±1.6) − 1.29 (±0.25) × time of day (h), Adj. *R*^2^ = 0.21, and *P* < 0.001 (SE are given in parentheses). Horizontal lines represent body mass of breeding birds at the breeding study site (71.3 ± 0.4 g), which we refer to as the population lean body mass in the main text.

We analyzed 493 observations of diurnally migrating nightjars distributed across 345 site-and-date combinations (Fig. 1A). All records were associated with a major sea crossing, which is congruent to observations in previous studies based on smaller samples of tracked birds that nightjars primarily are crepuscular and nocturnal animals (Table S1) (11, 14). Our data show that even open waters of moderate extension, such as the Baltic Sea, are regularly associated with flights that extend several hours into the day (Figs. 1A and 2 and Table S1). This is a surprising result because assuming that sea crossings are initiated at dusk, a nightjar flying at an airspeed of 10 m s^−1^ in still weather should fly more than 200 km (i.e. the approximate width of the Baltic Sea) in a 6-h nocturnal flight (18). Nonetheless, moderately wide waters also have the potential to become obstacles that influence a migrant's daily energy budget and survival prospects. Given the apparent risk of a fatal outcome during water crossings, migratory birds could choose to take detours that reduce the distance across water or to avoid it altogether (2, 19). However, the tracked nightjars did not make such detours and invariably crossed the Baltic and Mediterranean Seas both in autumn and spring (Fig. 1B and C).

The occurrence of nocturnal flights extending into day is traditionally explained either by the actual sea-crossing distance being too vast to be covered in one night's flight or by the presence of adverse weather during the flight (20, 21). Indeed, our GPS data revealed that open-water distance (OWD) (and hence risk of flight prolongations) was route specific (Fig. 1B and C). The variation in distance was most prominent during the spring passage of the Mediterranean Sea, where route choice resulted in an order of magnitude difference between the shortest and longest water crossings (Fig. 1D and E). Tracks including two successful GPS fixes during water-crossing nights allowed us to sample ground speed (Fig. 1F, mean = 16.35 m s^−1^, SD = 5.19). These recorded speeds were significantly higher than 10 m s^−1^ (i.e. the airspeed of a nightjar recorded by radar; one-sample *t* test = 11.15, *P* < 0.001, df = 82), indicating that the birds experience some wind assistance during these water crossings (18). We tested the effects of distance between GPS fixes, water distance covered prior to the first fix, and remaining water distance after the second fix (as proxies for groundspeed, relative crossing initiation timing, and barrier distance) on the probability of a diurnal flight event. We found that the probability of diurnal flights increased with OWD but decreased with higher ground speeds of the birds (Table 1). We did not detect any significant effect of water crossing initiation timing in our data. We also sampled wind data and calculated wind effects along the tracks to evaluate the influence of our approach to measure the birds’ ground speeds but this did not change the main results or conclusions (Tables S2–S8).

**Table 1.** Effects on the probability of diurnal flights of sea-crossing nightjars tracked by GPS.

	Estimate	SE	*z*-value	*P*
Intercept	3.9293	3.0060	1.307	0.1912
Open-water distance (km)	0.0507	0.0187	2.713	0.0067
Ground speed (m s^−1^)	−1.1432	0.4934	−2.317	0.0205
Completed barrier distance (km)	−0.0006	0.0618	−0.099	0.9210
Random intercept				
Group	Variance	SD		
Individual	3.392 × 10^−9^	5.824 × 10^−5^		

We applied a generalized linear mixed model with a binomial error distribution on a sample of 83 flights across 7 annual cycles from 24 individuals to analyze the effects of the barrier distance (the distance to arrival shore from the second GPS location), ground speed, which was the distance between the two GPS locations divided by sampling duration (2 h), and completed barrier crossing (the distance between the initiation point of the water crossing and the first GPS location) on the probability of diurnal flights. The completed barrier distance may be influenced by a combination of ground speed experienced by the birds during the evening before and by the timing of the water-crossing initiation. We found that the width of the barrier to cross increased the probability of diurnal flight events, while a higher ground speed reduced the likelihood of flights continuing into the daylight.

Body mass data of 95 nightjars examined just after completing a sea crossing show that birds caught in the early morning on average carry fuel reserves corresponding to about 12% of their approximate lean mass (set to 71.3 g, which was the mean mass of breeding birds in the study population that did not carry any fuel reserves; Fig. 2). This energy store could buffer for unexpected flight prolongations, but it decreases with time of day and may be depleted before such a prolonged flight is completed; migrating birds approach population lean mass by midday about 7 h later. To maximize realized flight distance given a limited amount of fuel, and thereby increase the likelihood of successful sea crossing, migrants should adopt behavioral responses to reduce transport costs. Many active flyers that normally exhibit continuous flapping flight can do that by switching to an intermittent flight where series of wing flaps are alternated with glides (Table S1 and Movie S1). Efficient gliders, such as nightjars (22) or other species with long and narrow wings, could save about 11–15% of their transport costs by flap-gliding (9, 23, 24).

**Fig. 3.**
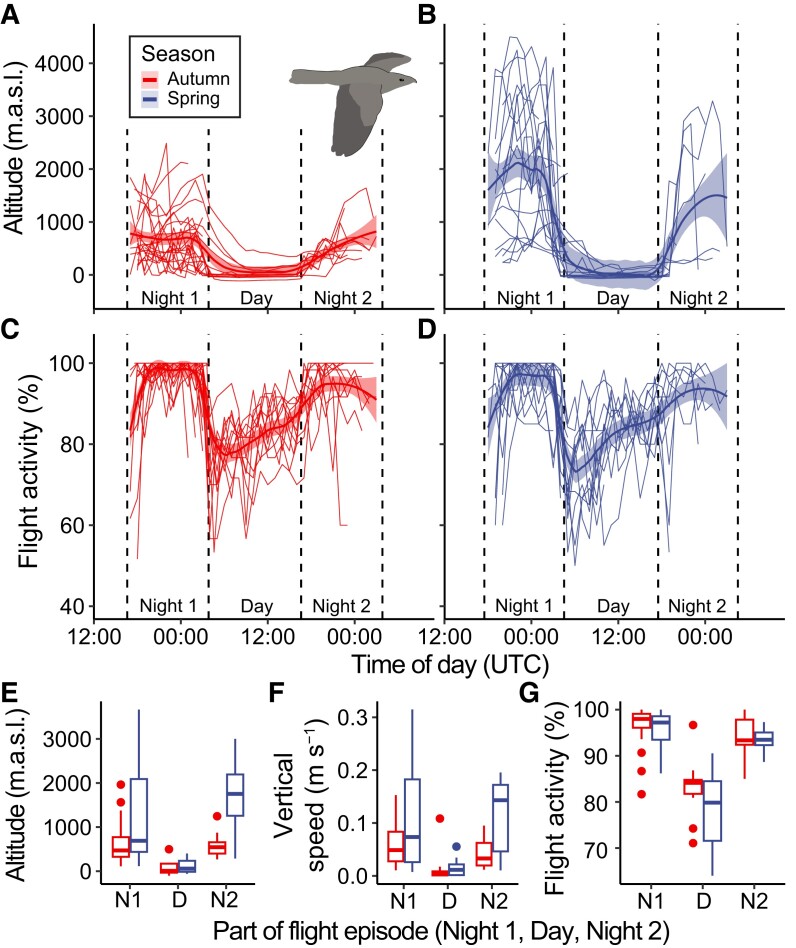
Flight characteristics of sea-crossing nightjars. A–D) Overview of flight altitude (A and B) and flight activity (C and D) relative to time of day during the crossing of the Mediterranean Sea in autumn (A and C) and spring (B and D) (see Fig. S1 for a corresponding description of the Baltic Sea crossing). Lines correspond to individual flights, and bold lines and ribbons represent loess smoothers and SE. Hatched vertical lines illustrate the approximate timing of sunrise and sunset. A, B) Flight altitude as derived from sampled ambient pressure by the MDL and presented as meters above sea level (m.a.s.l.). C, D) Level of flight activity calculated as the fraction of samples indicative of active flight. Means of flight altitude E), vertical speed F), and flight activity G) of flight episodes across Baltic and Mediterranean Seas relative to three time periods: first night (N1, *n* = 85); day (D, *n* = 30); and second night (N2, *n* = 19). Boxplots show the distribution of flight occurrences (line = median value, box = interquartile range [IQR], whiskers = 1.5 × IQR, and points = outliers). For each category left (red) and right (blue) boxplots correspond to autumn and spring.

Flight activity recorded by the MDL during sea crossings showed that about 35% (30 of 85 occasions) resulted in flights prolonged into daylight hours. The longest flights were recorded during the spring crossing of the Mediterranean Sea, where two birds were flying for 34 h after continuing throughout the day and into the following night (Fig. 3A and B). This corresponds to ∼1,000 km by a nightjar flying at 10 m s^−1^ and is comparable to the longest OWD recorded by GPS tags (Fig. 1C and F). The duration of flights associated with the crossing of the Baltic Sea was shorter (Fig. S1) likely due to the smaller distance covered during this passage (Fig. 1B and D). During flights involving a diurnal sea-crossing event, nightjars maintain a near-continuous flapping flight (mean 97.5 ± 2.71 SD % activity detections) throughout the first night. During daylight, the flight activity index dropped to 82.8 ± 4.76 SD %, characteristic for flap-gliding flight by diurnally flying nightjars (Table S1 and Movie S1). For 19 flight episodes that continued well into the following night, flight activity remained significantly lower (93.9 ± 3.83 SD %, *P* = 0.002, Table S9) than during the first night, suggesting that the birds continued to flap-glide throughout the remainder of the flight, although with a lower proportion of glides compared with flight in daylight. In tandem with flight activity reduction at dawn, the nightjars descended from their nocturnal flight altitudes and remained at lower altitudes throughout the day with minimal vertical movements (Fig. 3). A consistent low-altitude flap-gliding flight is accordant with field observations of nightjars almost skimming the water surface when approaching land (Table S1 and Movie S1), but here we show that this behavior is representative also for birds that later continued migration for over 20 h (while returning to higher altitudes around dusk; Fig. 3). This suggests that the diurnal low-altitude flight of nightjars is an adaptive behavior associated with the sea crossing regardless of their current fuel load (21). As nightjars exhibit an energy-conserving flap-glide flight style during diurnal flights, we predict that low-altitude flights over the sea surface will reduce flight costs further, as indicated by the increased glide fraction.

The occurrence of low-altitude flights could be explained by adaptations related to energy savings through four mechanisms, which may be alternated or combined depending on local weather conditions: (i) in situations when headwinds increase the cost of transport or when crosswinds cause drift, these negative effects can be reduced by flying in weaker winds close to the water surface (25, 26). However, if the negative effect of winds was a primary driver of diurnal flight altitude, we would expect birds to remain at their nocturnal levels under tailwind conditions, which was generally not the case during flights across water; (ii) by soaring on vertical gusts near wave crests, nightjars could alleviate the cost of transport associated with flapping flight (27); (3) by adopting a wave-slope soaring flight where they exploit local updrafts at surface gravity waves (28); and (4) by flying in ground effect, the aerodynamic cost associated with induced drag can be reduced considerably (29, 30). A mutual prerequisite for the three latter alternatives to be relevant is that the bird needs to move safely within a close vicinity to the sea surface, which may restrict surface-skimming flights to daylight hours. Accordingly, the nightjars that continued flying throughout the day returned to higher altitudes after dusk. Although the flight behavior of most self-powered terrestrial migrants over open waters is poorly known due to the methodological challenges to perform in situ studies, low-altitude flights are commonly observed among many small- to medium-sized seabirds in various wind conditions (31, 32).

Interestingly, the low diurnal flight altitude by nightjars is opposite to the pattern observed in two other long-distance avian migrants within the Palearctic–African migration system: the great snipe *Gallinago media* (33) and the great reed warbler *Acrocephalus arundinaceus* (34). Similar to nightjars, both species are active flyers that perform consistent altitude shifts between night and day, but the diurnal flights are at considerably higher altitudes than during night time, leading the authors to suggest three plausible explanations: (i) as the visible range increases with increasing altitude, flying high could allow the birds to identify safe landing sites; (ii) birds of prey that specialize on attacking migrating birds from above cause birds to fly high in daylight to evade predation risk; and (iii) extreme cruising altitudes improve passive heat transport to the surrounding air allowing the dissipation of added heat load due to increased solar radiation during daytime (33, 34). Neither of these potential explanations fit to the nightjar's behavior because (i) the visible range will be reduced when reaching the sea level; (ii) flying low over the seascape would make nightjars easy targets for aerial predators attacking from above (although this may primarily be a problem when approaching land; cf. Table S1); and (iii) given the negative temperature–altitude gradient, the ambient temperature is expected to be highest near ground level and considerably higher than at the 6,000–8,000 m above sea level diurnal cruising flight level reported for great reed warblers and great snipes (33, 34). Nonetheless, by using an energy-efficient flight behavior in daylight, nightjars likely reduce work-related heat production, thereby avoiding the risk of hyperthermia under heat stress caused by solar radiation (35).

There are many anecdotal observations of nocturnal bird migrants approaching coasts during early morning hours supporting the generality of the nightjars’ behavior. These terrestrial birds show an apparent effort to reach land by low flights just above the seascape to reach shelter on the shore. A common conception is that these presumed strugglers have been overtaken by unfavorable weather conditions during their sea crossing and that flying low is simply a way to escape the most taxing headwinds. Here, we show that sea-crossing events regularly result in diurnal low-altitude flights in an otherwise strictly nocturnal avian migrant. This, we argue, is a result of a migration tactic allowing birds to maximize flight range on a limited fuel load when crossing open water. Thus, this novel finding of adaptive altitude shifts by nightjars asks for a reconsideration of our understanding of flight strategies when land-birds migrate across seas.

## Materials and methods

### Study species

The European nightjar (henceforth nightjar) *C. europaeus* is a crepuscular and nocturnally active aerial insectivorous bird breeding across Europe and Asia with wintering areas in southern Africa (10). The flight behavior and migration of the population in SE Sweden (57.34°N, 16.21°E) have been studied since 2011 using a variety of dataloggers (11, 14, 36). Nightjars are energy-efficient flyers with long and slender wings (mean aspect ratio = 7.81, *n* = 9) and a relatively low wing loading (mean = 16.39 N m^−2^, *n* = 9). Consequently, they likely can perform vertical movements during migratory flights at relatively low costs, which may allow them to find altitudes with favorable wind conditions (22). Nightjars of the study population generally perform clockwise loop migrations between the European and African residence areas, likely due to large-scale trade wind patterns over Africa (36).

### Citizen science data

Records of nightjar observations were downloaded from three public databases (Data Sets S1–S3): eBird (https://ebird.org/data/download), the Swedish Species Observation System (SSOS, https://www.artportalen.se/), and the Norwegian Species Observation System (NSOS, https://www.artsobservasjoner.no/).

Observations of migrating nightjars were extracted from the SSOS and NSOS data sets. A subset of sites has been visited by birdwatchers more frequently, thus occasionally resulting in multiple reports of the same individual nightjar. To remove potential duplicates, we kept the minimum number of observations per site that were distinguishable, based on date, time of day, sex, and information in the “free text” column (e.g. “this bird was not the same individual observed by X”). This resulted in a final data set of 447 observations distributed among 323 site and date combinations (Fig. 1A, Table S1, and Data Sets S1 and S2).

The eBird data set does not contain information about whether the bird is migrating or not (37). To distinguish diurnal observations of sea-crossing nightjars from records of other scenarios (e.g. flushed birds roosting on ground), observations recorded over water or with a minimum distance of 50 m from the sea were extracted. From this subset, records with comments associated with migration or crossing the water were selected. This resulted in 46 observations distributed among 22 site and date combinations (Fig. 1A, Table S1, and Data Set S3).

All observations in the final data set were recorded near the sea or from boats and 237 observations on 141 occasions had additional comments referring to nightjars arriving from the sea or were flying over the sea surface (for example of such comments, see Table S1). While a single observation contained a comment about a (surprisingly) high altitude of the migrating birds, 14 comments refer to the low altitude of the birds (Table S1). One bird initiated a climbing flight (without any apparent reason noted by the observer), while other events of ascents were referred to as being interactions with other birds. Such interactions were typically attacks from falcons and gulls when nightjars approached the shoreline (Table S1).

### Body mass data

Birds were trapped during spring migration at Ottenby Bird Observatory, at the southern tip of Öland, SE Sweden (56.20°N, 16.40°E) between March 15 and June 15 every year following a standardized protocol since 1979 (38). Mist nets are activated ∼30 min before local dawn and are checked every 30 min until 11 AM (local time). At the same time, two permanent funnel-like Heligoland traps are checked. Trapped birds are taken to a processing room where they are ringed, examined, and measured before prompt release. Biometrics taken include wing length and body mass (using a spring balance to the nearest gram or an electronic balance to the nearest decimal gram).

For our analysis, we extracted birds from the database that had been trapped during the standardized spring-trapping scheme of the bird observatory and for which body mass data were recorded. This resulted in 95 records (Data Set S4).

We applied a linear regression model to examine the effect of time (local trapping hour) on recorded body mass.

### Tracking data, sampling

We used data from GPS tags deployed during 2015–2021 and MDL deployed during 2016–2021. The devices were deployed dorsally on the birds using a full-body harness. The total weight of logger and harness was between 1.9 and 2.1 g, corresponding to <3% of the lean mass of the birds (14).

GPS data included in this study were obtained from 2015 to 2022 from 26 individuals involving extended flights across water bodies (Data Set S5). The loggers were programmed to record two locations per night, at 9 PM and 11 PM (UTC), which allowed us to sample ground speed of sea-crossing birds during migration. Data coverage of the migration varies due to battery failure or other sampling failures. For the analyses of diurnal flight duration, we used a data set of 83 flights by 24 individuals. These were flights where both GPS fixes during the night involving the water crossing were successfully taken.

### Tracking data, extracting water-crossing flights

Periods covering the crossing of the Baltic and Mediterranean Seas were extracted. This data set was used to calculate the maximum distance of water crossing, ground speed of the birds, and estimated tailwind component along the track (Fig. S2). A flight step that included a water-crossing segment resulted in a minimum ground distance of *p*_A_ to *p*_B_ although the total flight distance (*p*_dep_ to *p*_arr_) may include distance both before and after the water crossing (Fig. S2). In each night flight, two GPS positions (*p*_1_ and *p*_2_) were taken near local midnight (9 PM and 11 PM, UTC). The distance between the two GPS fixes was used to derive ground speed (*V*_g_) during the water crossing. The locations (*p*_A_ and *p*_B_) are the intersections between the bird track and shorelines extracted from *Natural Earth* R package (39), using the intersect function in the *sf* R package (40). The distance between *p*_A_ and *p*_B_ along the track represents the OWD. Bird tracks were reconstructed by determining the great circle routes between consecutive GPS fixes, which may differ to a small extent from the actual route taken by the bird. We determined the timing of sea-crossing initiation at location *p*_A_ by dividing the distance between *p*_A_ and *p*_1_ with *V*_g_ and subtract the resulting duration from the timing of the first GPS position at *p*_1_. The expected arrival time of the bird at location *p*_B_ was determined by dividing the distance between *p*_2_ and *p*_B_ with *V*_g_ and adding the resulting duration to the timing of the second GPS position at *p*_2_. Timing of sunset at location *p*_A_ and sunrise at location *p*_B_ was derived from R package *suncalc* (41).

### Tracking data, analyzing wind data

As the daily sampling rate of the GPS devices was limited to two location recordings per night, the low temporal resolution will likely underestimate the distance the birds fly over open water and possibly also the flight duration over open water. More importantly, our estimates of the birds’ ground speed, and subsequent calculations of flight duration and timing of the water crossing, are based on a single measurement (the distance between the two nocturnal location recordings). Depending on how spatially correlated the realized wind support is along the track, this may increase the uncertainty of or bias our estimated parameters and influence our results. To explore the effects of such potential errors on our results, we sampled potential wind effects on an hourly basis along the track (see below) to calculate the correlation between different ground speeds estimated by the alternative approaches (Table S2) as well as the temporal correlations of wind effects during the open-water flights along the tracks (Tables S3 and S4). We also recalculated the parameters for ground speed and timing of water crossing given the following hypotheses regarding the flight behavior of nightjars (Tables S5–S8):

H1: Nightjars alter flight altitude regularly midflight to find the most supporting wind conditions. This hypothesis is supported by previous observations that nightjars from the study population regularly, and repeatedly, perform altitudinal movements during migratory flights (22).
H2: Nightjars continue to fly at or close to a specific altitude throughout the night, even though better tailwind conditions may occur at higher altitudes (42).

We used the mean flight altitude as recorded by the GPS as the flight altitude used by the nightjars in H2. As the processing of GPS positioning data can lead to negative altitude values or altitude values locked to the closest 250-m interval if no clear altitude solution is found within a reasonable time, we followed the procedure in Kearsley et al. (43) and excluded those altitude values. We therefore ended up with a sample of 62 sea-crossing tracks of 22 birds for H2 analyses.

We used the RNCEP R-package (44) to sample wind data from the NCEP/NCAR Reanalysis data set (45) provided by the NOAA/OAR/ESRL PSD, Boulder, CO, United States, from their web site at http://www.esrl.noaa.gov/psd/. Wind data were sampled along the track at linearly interpolated positions on an hourly basis at positions the birds would be if they maintained the ground speed measured between the two GPS recordings, using the Geosphere R-package (46). We analyzed winds at surface level and at four of the available pressure levels (i.e. 1,000, 925, 850, and 700 hPa) corresponding to approximate altitudes 100, 750, 1,500, and 3,000 m above sea level, commonly used by migrating nightjars in the region, including the GPS-tracked birds analyzed here (22). We used the function *NCEP.Airspeed* in RNCEP (44) to calculate the wind profit, with the assumption that the birds flew at a fixed airspeed and adjusting the heading (and thereby their ground speed during their flight) to maintain a preferred direction of migration (here along the recorded track). Airspeed was set to 10 m s^−1^, which is a radar measurement on the closely related red-necked nightjar *Caprimulgus ruficollis* (18). Finally, we calculated the harmonic mean of ground speeds along the tracks at the different flight altitudes according to H1 and H2 above. We tested both the full models (Tables S5 and S6) and models restricted to the significant variables in the original model (barrier distance and ground speed, Tables S7 and S8) when evaluating the H1 and H2.

### Tracking data, statistics

We applied a generalized linear mixed model with a binomial distribution to examine the effect of OWD (Fig. S2), *V*_g_, and completed barrier (distance between *p*_A_ and *p*_1_) on the probability of arriving to *p*_B_ in daylight using the *glmmTMB* R-package (47). Individual and deployment year were included as random intercepts, but due to model convergence problem, we only used individual in the final models. We evaluated that the residual distribution met the model assumptions using the *DHARMa* R-package (48). *R*^2^ values were extracted using the R-package *MuMIn* (49).

To evaluate if wind assistance at sea surface differed from the maximum wind assistance (H1) or at the wind assistance at the nocturnal flight altitude (H2), we applied a linear mixed model with derived ground speed as the dependent variable, altitude (surface, H1, and H2) as an independent factorial variable, and track id as a random intercept. We examined differences between groups using a post hoc test with Tukey approximation (50). We tested both the difference between groups in the first daylight hour (Table S10) and based on the mean wind assistance during the diurnal flights (Table S11). In both cases, the birds would experience a lower wind assistance at surface than at the altitude with maximum wind assistance (H1) whereas no significant difference was detected between surface level and the nocturnal flight altitude (H2).

### MDL data, sampling

Multisensory data included in this study were collected in 2016–2022 from 18 individuals (Data Sets S6 and S7) (11, 22). The loggers contained three sensors, an accelerometer, a pressure sensor, and a light sensor. Flight activity was sampled by the accelerometer in a sequence of 5 or 10 (depending on the version of MDL) 100-ms measurements of vertical acceleration (Fig. S3). To calculate the activity index used in this study, we divided the number of measurements indicating active flight with the total sample in the time interval. For example, a 75% activity means that 90 out of 120 samples indicated active flight in the 2016 and 2017 MDL versions and that 45 of 60 samples indicated active flight for logger versions from 2018 and onwards. The pressure sensor sampled ambient pressure at the location of the bird every hour (every 5 min during 2018–2019 and 2021–2022 seasons). Records of ambient pressure were translated to flight altitude above sea level by using the International Standard Atmosphere model (SA; International Organization for Standardization 1975: ISO 2533:1975):


$$z=\frac{T_{0}}{L}\;\left({\left(\frac{P_{0}}{P}\right)}^{\frac{LR_{0}}{g}}-1\right)$$

where *T*_0_ is temperature at sea level (assumed 288.15 K), *L* is the altitudinal lapse rate of temperature (−0.0065 K m^−1^), *P*_0_ is standard atmospheric pressure at sea level (1013.25 hPa), *P* is measured air pressure, *g* is acceleration due to gravity (9.81 m s^−1^), and *R*_0_ is the universal gas constant (287.053 J kg^−1^ K^−1^). The temperature compensated pressure sensor we used (Bosch Sensortech BMP280) had an absolute accuracy of ca. ±1 hPa, corresponding to ca. ±8 m. Each pressure sensor was factory calibrated with a unique set of individual calibration parameters. Altitude data used for analyses refer to the standard atmosphere-derived values, which are not corrected for local atmospheric conditions.

The MDL sampled light intensity during preprogrammed periods of the annual cycle and the data were used to geolocate the birds to reconstruct the positioning of the birds in relation to the annual cycle (51). For more details regarding the sampling routine of the MDL, see Norevik et al. (22).

### MDL data, extracting water-crossing flights

Sea-crossing flights were distinguished by interpolating the duration of migratory flight (as detected by the accelerometer) between known locations of the birds (from geolocation or breeding area), assuming a fixed ground speed. This approach does not allow us to reconstruct the exact flight path of the birds but make it possible to determine what flight episode that corresponds to the sea-crossing flight. Sea-crossing episodes involving extensive periods of sea surface skimming flights were used as validation as such long periods of low-altitude flights are only possible over open water. For the flight activity comparison between day and the first and second nights, we assigned hours (UTC) 9 PM to 3 AM to “night” and 6 AM to 4 PM to “day.”

### MDL data, statistics

We tested the categorical effect of the different time periods using a linear mixed effect model with mean flight activity as the dependent variable, the group as the independent variable, and individual bird as a random intercept (47). We evaluated that the residual distribution met the model assumptions using the *DHARMa* R-package (48). We examined differences between groups using a post hoc test with Tukey approximation (50).

## Supplementary Material

pgad225_Supplementary_Data

## Data Availability

All data needed to evaluate the conclusions in the paper are present in the paper and the supplementary materials.
