# Supplementary material for: Extremely low daylight sea-crossing flights of a nocturnal migrant
Source: PNAS Nexus. 2023 Jul 8;2(7):pgad225. doi: 10.1093/pnasnexus/pgad225 (PMC10355279; doi:10.1093/pnasnexus/pgad225)
Supplement: pgad225_Supplementary_Data [file pgad225_supplementary_data.zip › PNASNEXUS-PNASNEXUS-2023-00095R-s09.pdf]

PNAS nexus

# Supplementary Information for
Extremely low daylight sea-crossing flights of a nocturnal migrant

Gabriel Norevik, Susanne Åkesson, Anders Hedenström

Gabriel Norevik, Anders Hedenström
Email: gabriel.norevik@biol.lu.se, anders.hedenstrom@biol.lu.se

**This PDF file includes:**

Figures S1 to S3
Tables S1 to S11
Legends for Movie S1
Legends for Datasets S1 to S7

**Other supplementary materials for this manuscript include the following:**

Movie S1
Datasets S1 to S7

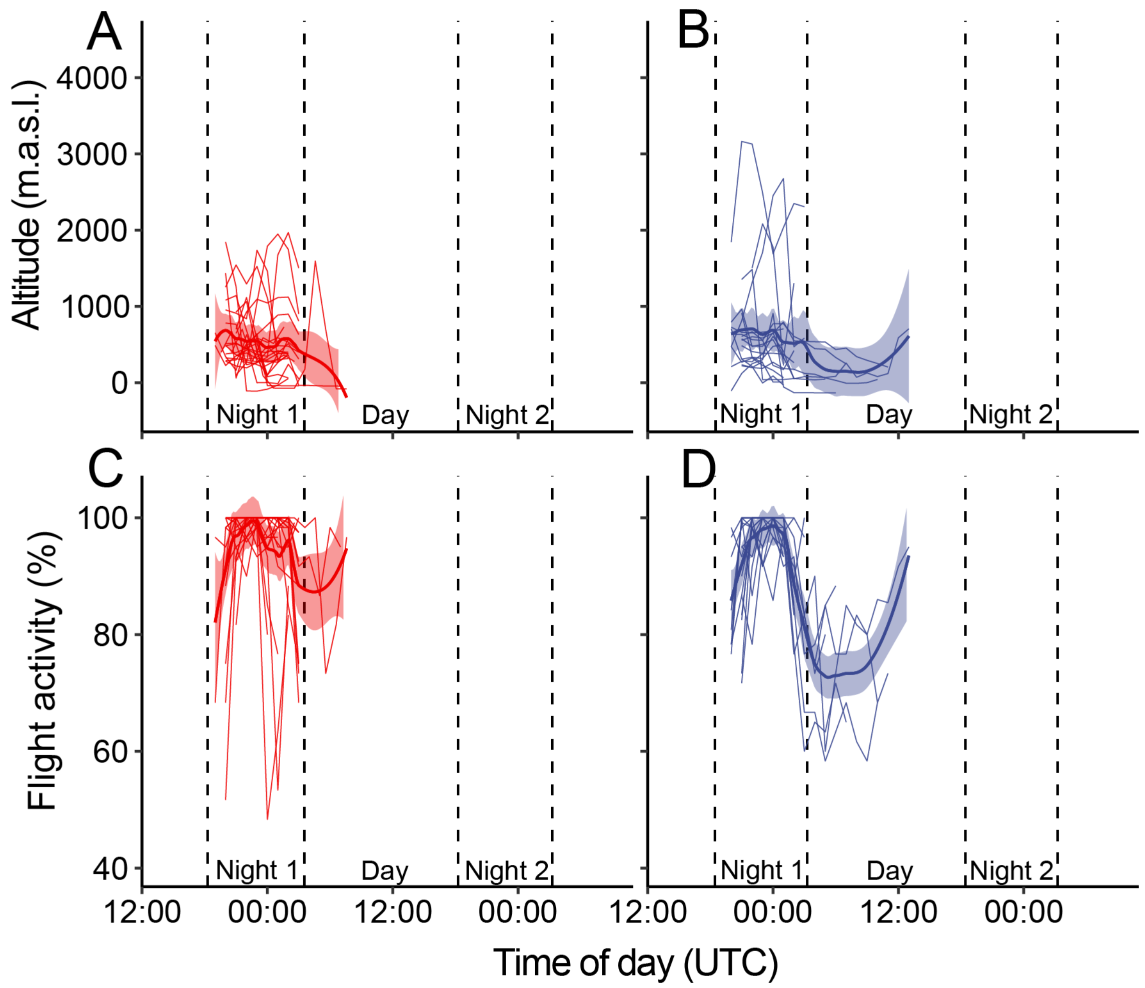


**Fig. S1. Flight characteristics of nightjars migrating across the Baltic Sea.**
Overview of flight altitude and flight activity relative to time of day during the crossing of the Baltic Sea in autumn (red) and spring (blue). Lines correspond to individual flights and bold lines and ribbons represent loess smoothers and SE. Hatched vertical lines illustrate the approximate timing of sunrise and sunset. (A, B) Flight altitude as derived from sampled ambient pressure by the MDL and presented as meters above sea level (m.a.s.l). (C, D) level of flight activity calculated as the fraction of samples indicative of active flight.

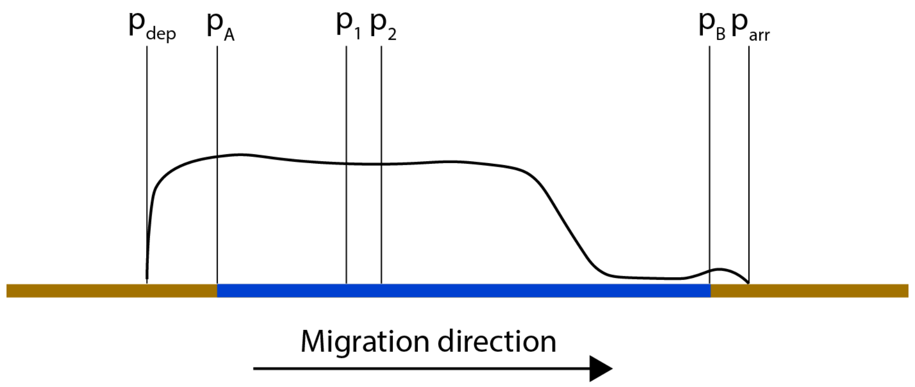


**Fig. S2. Conceptual presentation of a GPS-sampled water crossing of a nightjar.** Flight (smoothed line) is initiated at $p_{dep}$ and terminated at $p_{arr}$ and sampled around local midnight at positions $p_1$ and $p_2$. The intersection points between track and shoreline at $p_A$ and $p_B$ determine the open water distance (*OWD*) along the track, and thus the minimum distance of the flight across the water body.

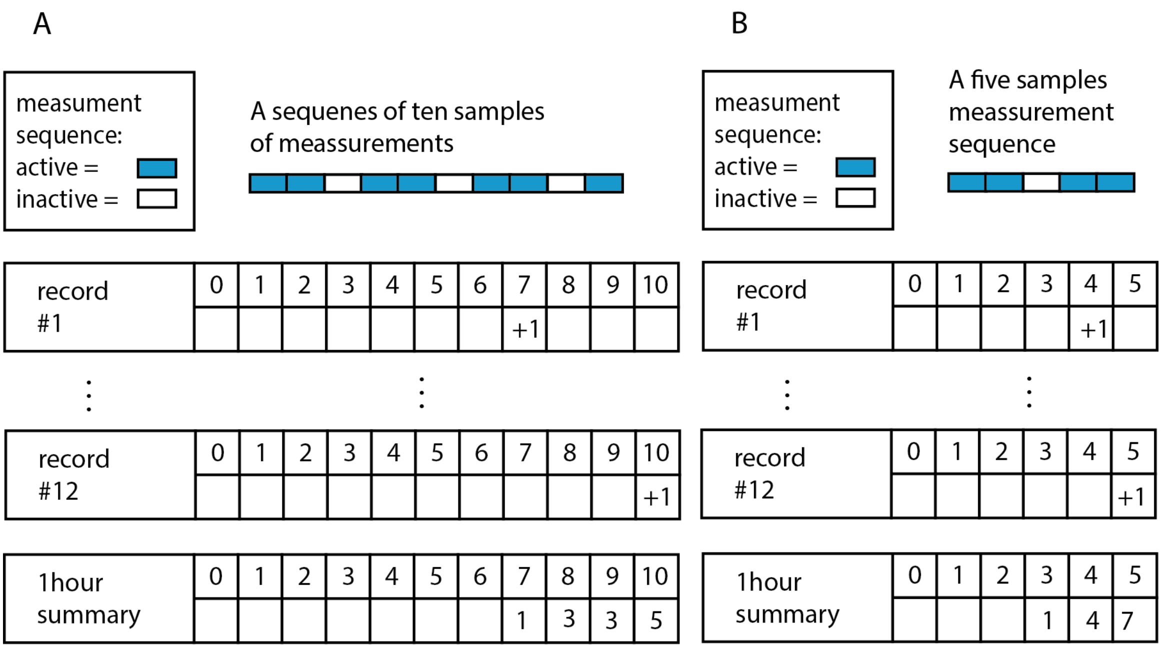


**Fig. S2. Conceptual illustration of the activity sampling program of the MDL deployed 2016 and 2017 (A) and 2018 to 2021.**

(A): Activity was sampled in a sequence of 10 measurements of 100 ms duration in 100 Hz with a 5 s sampling interval resulting in a record between 0 (acceleration varied less than ¼ g in each of the 10 measurements) and 10 (all measurements indicated flight activity). The procedure was repeated every 5 minute and was summarized every hour when 12 records were collected. For each hour a total of 120 measurements were taken. (B): As (A) but a sequence of 5 samples of measurements were taken resulting in a record between 0 (no registered activity) and 10 (all samples indicated flight activity). This sampling regime resulted in a total of 60 samples of measurements per hour.

**Table S1. A selection of observations from citizen science databases describing diurnal sea-crossing migration events of nightjars.**

Comments are citations from the observer's 'free-text' comment regarding each recorded observation.

| Date | Site | Country | Comment |
|---|---|---|---|
| 1984-09-27 | Gulf of Oman | Oman | Female entered hangar [of the ship] immediately [when] door opened at 08:15 (local time]. Hangar active so [it] left. Male flew behind the ship. |
| 1995-06-17 | Limassol to Port Said ferry crossing | Cyprus | Seen flying low over the sea in the late afternoon but while still daylight. Obviously a migrating bird. |
| 2006-06-08 | Hammarö | Sweden | Came in low over the water and was attacked by two Eurasian hobbies [*Falco subbuteo*][1] |
| 2008-05-10 | Southern Öland | Sweden | High altitude, attacked by jackdaws [*Coloeus monedula*][1] |
| 2013-05-04 | Southern Öland | Sweden | Was discovered thanks to an attacking Herring gull [*Larus argentatus*]. The nightjar then ascent rather high. When the encounter was over, it descended to wave-top level and was then impossible to observe.[1] |
| 2014-05-22 | Lista | Norway | Came in from the sea ... Hit by a peregrine falcon [*Falco peregrinus*] close to land and seconds later engulfed by a great black-backed gull [*Larus marinus*].[2] |
| 2014-05-22 | Lista | Norway | Came in from the sea ... Ascended when the peregrine [falcon] was approaching and continued migrating towards north-west.[2] |
| 2014-06-01 | Southern Öland | Sweden | Came in from south, encountered by a herring gull. It then ascended and should have reached the point [of the island] at a rather high altitude, which is not common. The migration is usually conducted just above the sea-surface.[1] |
| 2014-06-05 | Southern Öland | Sweden | Was discovered ... when it was attacked by gulls. The nightjar ascended to a rather high altitude and continued migrating towards north/northeast, sometimes in gliding flight ...[1] |
| 2014-06-12 | Southern Öland | Sweden | ... Passed the observation point and continued north between the cars on the parking lot ...[1] |
| 2014-09-20 | Khor Kalba Pelagic | United Arab Emirates | Flying south low over the water, 3 km from land. Immature. |
| 2015-05-22 | Fårö | Sweden | Followed the boat up-close for a while before heading towards land.[1] |
| 2016-05-11 | Hönö | Sweden | Was discovered 9:55 [local time] when it was about 500 m out over the sea. Struggled in the strong easternly wind, and with a few harassing gulls.[1] |
| 2016-05-12 | Southern Öland | Sweden | Was discovered early migrating towards northwest. ... Was observed for 15 minutes ... |

| | | | |
|---|---|---|---|
| | | | Presumably not exhausted, just annoyed by encounters with gulls.[1] |
| 2016-06-30 | Khor Kalba Pelagic | United Arab Emirates | In straight flight, 1 meter above the sea. Presumably migrating bird between Iran and Arabia. 4 seen in the morning by [our captain] Abdallah, and 10 the day before, in similar conditions. |
| 2019-06-11 | Kungsbacka | Sweden | It came in 17:40 [local time] ... Bullied by gulls. Presumably did not land on the island but was later seen migrating east.[1] |
| 2020-05-12 | Southern Öland | Sweden | All [three] came in from the sea at a surprisingly high altitude, in the strong northern [head]wind. Two descended and landed ... One continued migrating...[1] |
| 2020-05-26 | Southern Öland | Sweden | ... In addition, a mystery bird, closer, that presumably was a nightjar although it flew more like a falcon, actively, with just one gliding event over the laps of several minutes.[1] |
| 2020-05-29 | Listerby | Sweden | Came from south and migrated persistently in over land. ... Sparse flocks of 5, 4, 3, 3, and 7*2 ex as well as 18 single birds [were observed]. Weather was clear with a weak wind from northeast/east.[1] |
| 2021-05-21 | Southern Öland | Sweden | ... came in low from southwest. Near land it ascended while flying in circles for some reason. Could not confirm any attacks, perhaps concerned anyway.[1] |
| 2021-06-05 | Southern Öland | Sweden | Came in low over the sea. A peregrine falcon chased [the nightjar] but it seems to have survived.[1] |

1. Authors' translation from Swedish.
2. Authors' translation from Norwegian.

**Table S2. Correlation between ground speed estimates derived from GPS data and wind data.**

We calculated correlation coefficient (*r*) between ground speed estimates based on the distance between two GPS locations and based on wind effect (according to H1 and H2 described in the main text) along the track during the same time period (between 21:00 and 23:00 hours UTC). For each correlation Pearson's correlation coefficient is provided along with confidence interval (*CI*), number of degrees of freedom (*df*) and *p*.

| **Correlation** | *r* | *CI* | *df* | *p* |
|---|---|---|---|---|
| GPS – H1 | 0.79 | 0.70-0.86 | 81 | < 0.001 |
| GPS – H2 | 0.68 | 0.52-0.79 | 60 | < 0.001 |

**Table S3. Temporal correlation between ground speed estimates derived from wind data sampled along the track of the open water crossing at the altitude of maximum wind support (H1).**

We calculated correlation coefficient (*r*) of ground speeds estimates based on wind effects (according to H1 described in the main text) at 22:00 UTC and every other hour between 16:00 and 07:00 UTC. For each correlation Pearson's correlation coefficient is provided along with confidence interval (*CI*), number of degrees of freedom (*df*) and *p*.

| **Correlation** | *r* | *CI* | *df* | *p* |
|---|---|---|---|---|
| 22 – 16 | 0.88 | 0.69-0.96 | 15 | < 0.001 |
| 22 – 17 | 0.88 | 0.75-0.95 | 25 | < 0.001 |
| 22 – 18 | 0.78 | 0.64-0.87 | 48 | < 0.001 |
| 22 – 19 | 0.71 | 0.55-0.82 | 55 | < 0.001 |
| 22 – 20 | 0.71 | 0.55-0.82 | 55 | < 0.001 |
| 22 – 21 | 0.99 | 0.98-0.99 | 56 | < 0.001 |
| 22 – 22 | 1.00 | 1.00-1.00 | 73 | < 0.001 |
| 22 – 23 | 0.87 | 0.80-0.92 | 73 | < 0.001 |
| 22 – 00 | 0.84 | 0.76-0.90 | 71 | < 0.001 |
| 22 – 01 | 0.74 | 0.58-0.85 | 45 | < 0.001 |
| 22 – 02 | 0.77 | 0.57-0.88 | 29 | < 0.001 |
| 22 – 03 | 0.71 | 0.41-0.87 | 20 | < 0.001 |
| 22 – 04 | 0.78 | 0.49-0.91 | 16 | < 0.001 |
| 22 – 05 | 0.56 | 0.04-0.84 | 12 | 0.037 |
| 22 – 06 | 0.52 | -0.16-0.87 | 8 | 0.11 |
| 22 – 07 | 0.45 | -0.31-0.86 | 7 | 0.23 |

**Table S4. Temporal correlation between ground speed estimates derived from wind data sampled along the track of the open water crossing at the flight altitude recorded by GPS (H2).**

We calculated correlation coefficient (*r*) of ground speeds estimates based on wind effects (according to H2 described in the main text) at 22:00 UTC and every other hour between 16:00 and 05:00 UTC. For each correlation Pearson's correlation coefficient is provided along with confidence interval (*CI*), number of degrees of freedom (*df*) and *p*.

| **Correlation** | *r* | *CI* | *df* | *p* |
|---|---|---|---|---|
| 22 – 16 | 0.66 | 0.14-0.89 | 10 | 0.020 |
| 22 – 17 | 0.73 | 0.40-0.89 | 16 | < 0.001 |
| 22 – 18 | 0.65 | 0.40-0.81 | 33 | < 0.001 |
| 22 – 19 | 0.53 | 0.26-0.72 | 37 | < 0.001 |
| 22 – 20 | 0.54 | 0.27-0.72 | 38 | < 0.001 |
| 22 – 21 | 0.99 | 0.98-0.99 | 56 | < 0.001 |
| 22 – 22 | 1.00 | 1.00-1.00 | 52 | < 0.001 |
| 22 – 23 | 0.75 | 0.61-0.85 | 52 | < 0.001 |
| 22 – 00 | 0.76 | 0.61-0.85 | 51 | < 0.001 |
| 22 – 01 | 0.78 | 0.59-0.88 | 31 | < 0.001 |
| 22 – 02 | 0.60 | 0.25-0.81 | 21 | 0.002 |
| 22 – 03 | 0.44 | -0.06-0.76 | 15 | 0.08 |
| 22 – 04 | 0.36 | -0.24-0.76 | 11 | 0.23 |
| 22 – 05 | 0.09 | -0.57-0.68 | 8 | 0.81 |

**Table S5. Effects on the probability of diurnal flights of sea-crossing nightjars tracked by GPS, full model with ground speed estimated according to H1.**

We applied a generalized linear mixed model with a binomial error distribution on a sample of 83 flights from 24 individuals to analyze the effects of the barrier distance (the distance to arrival shore from the second GPS location), ground speed, which was derived according to H1, and completed barrier crossing (the distance between the initiation point of the water crossing and the first GPS location) on the probability of diurnal flights.

| | **Estimate** | **SE** | **z value** | **p** |
|---|---|---|---|---|
| Intercept | -1.5255 | 2.0001 | -0.763 | 0.4456 |
| Open water distance (km) | 0.0244 | 0.0114 | 2.143 | 0.0032 |
| Ground speed (m $s^{-1}$) | -0.3403 | 0.2164 | -1.573 | 0.1157 |
| Completed barrier distance (km) | -0.0047 | 0.0660 | -0.717 | 0.4731 |

Random intercept

| Group | Variance | SD |
|---|---|---|
| Individual | 0.6282 | 07926 |

**Table S6. Effects on the probability of diurnal flights of sea-crossing nightjars tracked by GPS, full model with ground speed estimated according to H2.**

We applied a generalized linear mixed model with a binomial error distribution on a sample of 62 flights from 22 individuals to analyze the effects of the barrier distance (the distance to arrival shore from the second GPS location), ground speed, which was derived according to H2, and completed barrier crossing (the distance between the initiation point of the water crossing and the first GPS location) on the probability of diurnal flights.

| | **Estimate** | **SE** | **z value** | **p** |
|---|---|---|---|---|
| Intercept | -2.5734 | 1.6770 | -1.535 | 0.1249 |
| Open water distance (km) | 0.0193 | 0.0053 | 3.633 | 0.0003 |
| Ground speed (m $s^{-1}$) | -0.1677 | 0.1363 | -1.230 | 0.2186 |
| Completed barrier distance (km) | -0.0053 | 0.0052 | -1.034 | 0.3012 |

Random intercept

| Group | Variance | SD |
|---|---|---|
| Individual | $7.098 \times 10^{-9}$ | $8.425 \times 10^{-5}$ |

**Table S7. Effects on the probability of diurnal flights of sea-crossing nightjars tracked by GPS, restricted model with ground speed estimated according to H1.**

We applied a generalized linear mixed model with a binomial error distribution on a sample of 83 flights across seven annual cycles from 24 individuals to analyze the effects of the barrier distance (the distance to arrival shore from the second GPS location), ground speed, which was derived according to H1.

| | **Estimate** | **SE** | **z value** | **p** |
|---|---|---|---|---|
| Intercept | -1.2922 | 1.8057 | -0.716 | 0.4742 |
| Open water distance (km) | 0.0224 | 0.0056 | 3.999 | <0.001 |
| Ground speed (m $s^{-1}$) | -0.341 | 0.1632 | -2.088 | 0.037 |

Random intercept

| Group | Variance | SD |
|---|---|---|
| Individual | $8.925 \times 10^{-7}$ | $9.447 \times 10^{-4}$ |

**Table S8. Effects on the probability of diurnal flights of sea-crossing nightjars tracked by GPS, restricted model with ground speed estimated according to H2.**

We applied a generalized linear mixed model with a binomial error distribution on a sample of 62 flights across seven annual cycles from 22 individuals to analyze the effects of the barrier distance (the distance to arrival shore from the second GPS location), ground speed, which was derived according to H2.

| | **Estimate** | **SE** | **z value** | **p** |
|---|---|---|---|---|
| Intercept | -2.4049 | 1.6333 | -1.472 | 0.1409 |
| Open water distance (km) | 0.0185 | 0.0050 | 3.676 | 0.0002 |
| Ground speed (m $s^{-1}$) | -0.1977 | 0.1322 | -1.495 | 0.1350 |

Random intercept

| Group | Variance | SD |
|---|---|---|
| Individual | $6.061 \times 10^{-9}$ | $7.785 \times 10^{-5}$ |

**Table S9. Comparison of mean flight activity between first night (Night 1), day (Day)and second night (Night 2).**

We applied a general linear mixed model with a gaussian error distribution on a sample of 19 flights that continued well into the second night with flight activity as a dependent variable, flight episode as independent factorial variable and track id as a random intercept. We applied a Tukey approximation for multiple comparison of groups.

| Linear hypothesis | Estimate | SE | z value | p |
|---|---|---|---|---|
| First night – Day | 0.1467 | 0.0104 | 14.135 | <0.0001 |
| Second night – Day | 0.1109 | 0.0104 | 10.681 | <0.0001 |
| Second night – First night | -0.0359 | 0.0104 | -3.453 | 0.0016 |

**Table S10. Comparison of wind influenced ground speeds at three different flight altitudes in the first hour of diurnal flight.**

Surface (surface level), H1 (altitude with max wind support) and H2 (altitude corresponding to the measured altitude according to H2). We applied a general linear mixed model with a gaussian error distribution on a sample of 17 tracks that continued into day with ground speed as a dependent variable, flight altitude as independent factorial variable and track id as a random intercept. We applied a Tukey approximation for multiple comparison of groups.

| **Linear hypothesis** | **Estimate** | **SE** | **z value** | **p** |
|---|---|---|---|---|
| H2 – H1 | -1.2305 | 0.4550 | -2.705 | 0.018 |
| Surface – H1 | -1.9193 | 0.3980 | -4.822 | <0.001 |
| Surface – H2 | -0.6887 | 0.4550 | -1.514 | 0.284 |

**Table S11. Comparison of the average ground speeds at three different flight altitudes during daylight.**

Surface (surface level), H1 (altitude with max wind support) and H2 (altitude corresponding to the measured altitude according to H2. We applied a general linear mixed model with a gaussian error distribution on a sample of 17 tracks that continued into day with ground speed as a dependent variable, flight altitude as independent factorial variable and track id as a random intercept. We applied a Tukey approximation for multiple comparison of groups.

| Linear hypothesis | Estimate | SE | z value | p |
|---|---|---|---|---|
| H2 – H1 | -1.4412 | 0.4545 | -3.171 | 0.004 |
| Surface – H1 | -1.9809 | 0.3975 | -4.984 | <0.001 |
| Surface – H2 | -0.5397 | 0.4545 | -1.187 | 0.460 |

**Movie S1 (separate file). Video recording of two nightjars as they approach land during their spring migration across the Baltic Sea.**
Both individuals illustrate a low-altitude flap-gliding flight typically exhibited by migrating nightjars observed flying over water in daytime. Video courtesy Fredrik Lennartsson.

**Dataset S1 (separate file).**
Data file in plain text with observations downloaded from eBird contain a data identifier, information about the number of birds observed, sex/age location (in latitude longitude), date and time for the observation and a comment about the observation.

**Dataset S2 (separate file).**
Data file in plain text with observations downloaded from the Norwegian Species Observation System contain information regarding the number of individuals recorded and their age/sex, activity (e.g. migrating), location (in national east and north coordinates), and start and end datetimes of the observation.

**Dataset S3 (separate file).**
Data file in plain text with observations downloaded from the Swedish Species Observation System contain information regarding the number of individuals recorded and their age/sex, activity (e.g. migrating), location (in national east and north coordinates), and start and end datetimes of the observation.

**Dataset S4 (separate file).**
Data file in plain text with body mass data from migrating nightjars contains timestamps (UTC + 2), bird id and recorded body mass for birds trapped at Ottenby Bird Observatory, SE Sweden (56.20° 16.40°).

**Dataset S5 (separate file).**
Data file in plain text with global positioning data from sea-crossing nightjars contains recorded timestamps (UTC) along with locations (latitude and longitude) for each tag and bird.

**Dataset S6 (separate file).**
Data file in plain text containing multisensory data: For each timestamp (UTC) the distribution of 12 activity registrations is presented in columns Act0 to Act10 that represent a range of activity levels given a predefined sampling routine. See, methods and supplement in the related work. Column "check" is the sum of all activity counts and should be 12 if sampling worked as intended. The file also contains the recorded temperature in °C and barometric pressure in mbar.

**Dataset S7 (separate file).**
Data file in plain text containing multisensory data: For each timestamp (UTC) the distribution of 12 activity registrations is presented in columns Act0 to Act5 that represent a range of activity levels given a predefined sampling routine. See, methods and supplement in the related work. Column "check" is the sum of all activity counts and should be 12 if sampling worked as intended. The file also contains the recorded temperature in °C and barometric pressure in mbar.
